# The Trained Nurse

**Published:** 1890-02

**Authors:** 


THE TRAINED NURSE. Vol. III, No. 6, of "The Trained Nurse."

A journal consecrated to those who minister to the sick and the suffering comes to us with a request that we make known the endeavor to form an organization of "Trained Nurses for New York State, which will be made the basis of a national organization."

We are heartily at one with the movers of this association, as we believe it will be of mutual interest to both physicians and nurses. *The Trained Nurse* is a journal published by the Lakeside Publishing Co., Buffalo, N. Y. If all its numbers are as valuable as its holiday number we would advise our readers to invest the $1.50 yearly necessary to obtain it.

